# Identify BCAT1 plays an oncogenic role and promotes EMT in KIRC via single cell RNA-seq and experiment

**DOI:** 10.3389/fonc.2024.1446324

**Published:** 2024-09-11

**Authors:** Shiqing Li, Yinsheng Guo, Guanhua Zhu, Lu Sun, Feng Zhou

**Affiliations:** Department of Urology, The First Affiliated Hospital of Soochow University, Suzhou, China

**Keywords:** BCAT1, renal cell carcinoma, EMT, biomarker, tumor immune microenvironment

## Abstract

**Background:**

Kidney renal clear cell carcinoma (KIRC) is a major subtype of renal cell carcinoma with poor prognosis due to its invasive and metastatic nature. Despite advances in understanding the molecular underpinnings of various cancers, the role of branched-chain amino acid transferase 1 (BCAT1) in KIRC remains underexplored. This study aims to fill this gap by investigating the oncogenic role of BCAT1 in KIRC using single-cell RNA-seq data and experimental validation.

**Methods:**

Single-cell transcriptomic data GSE159115 was utilized to investigate potential biomarkers in KIRC. After screening, we used BCAT1 as a target gene and investigated its function and mechanism in KIRC through databases such as TCGA-GTEx, using genome enrichment analysis (GSEA), genome variation analysis (GSVA), gene ontology (GO) and Kyoto Encyclopedia of the Genome (KEGG). BCAT1 expression was detected in clinical tissue samples using Western Blotting (WB) and immunohistochemical (IHC) staining techniques. We established cell lines stably overexpressing and knocking down BCAT1 and performed WB, qRT-PCR, cell scratch assay and transwell assay.

**Results:**

BCAT1 was highly expressed in KIRC and was associated with disease prognosis and TME. Patients with mutations in the BCAT1 gene had shorter overall survival (OS) and disease-free survival (DFS). patients with high BCAT1 expression had shorter OS, progression-free interval (PFI), and disease-specific survival (DSS). GSEA showed that BCAT1 was significantly enriched in epithelial mesenchymal transition (EMT). Bioinformatics analysis and WB and IHC staining showed that BCAT1 expression was higher in KIRC than in paracancerous tissues. *In vitro* experiments confirmed that BCAT1 in KIRC cells may promote EMT affecting its invasion, migration. We constructed a protein interaction network (PPI) to hypothesize proteins that may interact with BCAT1. Single-sample gene set enrichment analysis (ssGSEA) revealed the immune infiltration environment of BCAT1. Furthermore, hypomethylation of the BCAT1 promoter region in KIRC may contribute to disease progression by promoting BCAT1 expression.

**Conclusion:**

BCAT1 promotes KIRC invasion and metastasis through EMT and has prognostic predictive value and potential as a biomarker. It may become a novel biomarker.

## 1 Background

Branched-chain amino transferases 1 (BCAT1) is a cytoplasmic isoform of branched-chain amino acid transferase found in humans and a variety of organisms (1). Branched-chain amino acids (BCAAs) include valine, leucine, and isoleucine. They serve as a nitrogen source for the formation of macromolecules such as nucleotides and are also essential for cancer cell growth. The metabolic flexibility of cancer cells is determined by their ability to reprogram synthetic and catabolic pathways through altered gene expression programs and intercellular interactions in the tumor microenvironment (2, 3). In many cancers, enzymes with amino acid catabolic functions are highly expressed. These enzymes not only provide cellular energy and metabolites for anabolic processes, but also promote immune escape of cancer cells. Intracellularly, BCAA is catalyzed by branched-chain amino acid transaminase (BCAT) to branched-chain α-keto acids (BCAA), including α-keto-β-methylglutaric acid (KMV), α-ketoisocaproic acid (KIC), and α-ketoisovaleric acid (KIV), while the amino group of BCAA is transferred to α-ketoglutaric acid (α-KG) to produce glutamate. BCAA can eventually be degraded to acetyl coenzyme A and succinate coenzyme A, which enter the tricarboxylic acid (TCA) cycle (4–7). The two isoforms of BCAT, BCAT1 and BCAT2, are located in the cytoplasm and mitochondria, respectively, and metabolic reprogramming of BCAA involving BCAT1 alters the levels of important metabolites, including BCAA itself, α-KG, and glutamate, which are used to produce nutrients and essential substances, activate signaling pathways, shape the epigenetic landscape, and enhance drug resistance, ultimately leading to a rapid increase in cancer cell survival and growth (7). We have reviewed the literature and found that high expression of BCAT1 is associated with acute and chronic myeloid leukemia (4, 8, 9), glioma (10–12), hepatocellular carcinoma (1, 10, 13–15), pancreatic cancer (16), gastric cancer (17, 18), colorectal cancer (19–21), melanoma (22), breast cancer (23–27), uroepithelial cancer (28), prostate cancer (29), osteosarcoma (30), ovarian cancer (31), endometrial cancer (32), nasopharyngeal cancer (33), esophageal squamous carcinoma (34), and non-small-cell lung cancer (35–37) have been implicated in the progression and prognosis of these cancers. Currently, BCAT1 has been proposed as a prognostic marker for a variety of cancer types and has multiple functions in cancer growth and progression. However, the role and mechanism of BCAT1 in renal cancer is not clear.

Kidney cancer (KC) is the sixth most common cancer in men and the tenth most common in women, accounting for 5% and 3% of all cancer cases, respectively (38, 39). Although many epidemiologically reported data refer to KC, histologically identified renal cell carcinoma (RCC) accounts for the vast majority (90%) of KC cases, with kidney renal clear cell carcinoma (KIRC) accounting for more than 70% of RCC (40). The leading cause of death in cancer patients is reported to be metastasis, accounting for more than 90% of cases (41).

Epithelial-to-mesenchymal transition(EMT) affects the ability of tumor cells to invade and metastasize through multiple pathways and is an important process in the transition of malignancy from early to advanced stages, whereby fully differentiated cells lose their cell polarity and intercellular adhesion properties and instead acquire a migratory and invasive mesenchymal phenotype (42, 43).

## 2 Methods

### 2.1 Data collection

The gene mRNA expression data and clinicopathological information of TCGA and GTEx were downloaded from the UCSC Xena database (http://xenabrowser.net/datapages/) for analysis. The single-cell sequencing dataset GSE159115 from KIRC was used to download the single-cell level expression data matrix containing single-cell level cell type annotations via TISCH2 (44) (http://tisch.comp-genomics.org/). Protein interaction networks were constructed using GeneMANIA (https://genemania.org/) and STRING (https://cn.string-db.org/).

### 2.2 Processing and analysis of single-cell RNA sequencing data

The single-cell expression matrix of the KIRC single-cell dataset GSE159115 was loaded and created as “SeuratObject” via the “Seurat” package (45), which annotated 27669 single cells with cell type based on the cell meta information therein. The 2000 high-variable genes were filtered using the “FindVariableFeatures” function and the “ScaleData” function. And based on the highly variable genes, PCA was performed on single cells using “RunPCA” function to reduce the dimensionality, check the PCA binning results and select the principal components. Subsequently, the KIRC single-cell data were clustered and analyzed using the Louvain algorithm, and the data were nonlinearly dimensionalized using the “Uniform Manifold Approximation and Projection (UMAP)” function (46). Malignant cells from KIRC in GSE159115 were then extracted using the subset function in the Seurat package, and cell subclustering was performed using the “FindNeighbors” and “FindClusters” functions, followed by UMAP nonlinear dimensionality reduction visualization in the same manner as described above. Marker gene analysis was performed for subclustering of Malignant cells by the “FindAllMarkers” function, and Marker genes were analyzed using the Wilcoxon algorithm in a selected group vs rest manner. The R package Monocle2 was used for the proposed chronological analysis of subclusters of Malignant cells (47), and the cells were sorted using subcluster marker genes to determine the gene expression patterns among Malignant differentiation trajectories as a whole, and the differentiation trajectory-related genes were searched by the “differentialGeneTest” function.

### 2.3 The cBioPortal

cBioPortal is an online tool for tumor genomics data analysis. We selected Kidney Renal Clear Cell Carcinoma (TCGA, Firehose Legacy) as the study dataset in the cBioPortal database, which contains 538 KIRC samples with mutations, copy number variants and mRNA expression data with survival analysis, clinical characterization, etc.

### 2.4 GO and KEGG pathway enrichment analyses

Through Gene Ontology (GO) analysis we obtained information on the biological process, cell composition and molecular function related to BCAT1 (48). In addition, we used the Kyoto Encyclopedia of Genes and Genomes (KEGG) to analyze the role of BCAT1 in metabolic pathways (49). We used the R package “ClusterProfiler” for GO and KEGG enrichment analysis of the biological functions of BCAT1 in KIRC (50).

### 2.5 GSEA and GSVA

We performed the analysis using the Gene Set Enrichment Analysis (GSEA) package (4.1.0) for discovering specific biological processes and disease-associated biological pathways or gene sets associated with BCAT1 gene differential expression in the database.

Gene Set Variation Analysis (GSVA) which is a non-parametric and unsupervised algorithm was used for Bulk RNA-seq data (TCGA) using the “gsva” algorithm in the GSVA package to quantify the hallmark of each sample from KIRC gene sets for each sample of KIRC (51). The “limma” package also was used to analyze the differential genes between high and low enrichment scores (52). For scRNA-seq data, the “gsva” algorithm was performed to calculate the enrichment scores of hallmark gene sets in each cell and count the differences in the enrichment scores of the hallmark gene sets between different cell types and different differentiation trajectories of malignant cells.

### 2.6 TISIDB

An integrated repository portal for tumor-immune system interactions (TISIDB) integrates multiple data types including PubMed database, transcriptomic and clinical data from TCGA, exomics and RNA sequencing datasets from immunotherapy patient cohorts, and we used it to analyze the relationship between BCAT1 and lymphocyte infiltration, immunomodulators, chemokines, and immune subtypes.

### 2.7 UALCAN

The University of Alabama at Birmingham Cancer Data Analysis Portal (UALCAN) is an interactive public database for analyzing cancer histology data.

We analyzed the relationship between BCAT1 mRNA expression levels, promoter region methylation levels, and clinicopathological KIRC information via the UALCAN website by selecting the “TCGA” section.

### 2.8 Construction of prognostic signature

The R packages “survival” and “survminer” were used for Kaplan-Meier prognostic analysis of the high and low BCAT1 expression groups in the TCGA-KIRC dataset. The R package “timeROC” was utilized to evaluate the AUC values of the ROC curves over time. To obtain a more accurate nomogram, the R package “rms” was used to combine pathological staging with risk scores in order to further improve the predictive efficiency of risk scores (53).

### 2.9 Cell culture and specimens

The 786-o(RRID: CVCL_1051), 769-p(RRID: CVCL_1050) and 293T(RRID: CVCL_0063) cell lines used in this experiment were purchased from the cell bank of the Chinese Academy of Sciences and identified by the STR test. The cells were cultured at 37°C in a 5% CO_2_ humidified incubator using RPMI-1640 and DMEM/high glucose medium containing 10% FBS (Shanghai Yuanpei Biotechnology Co., Ltd., China). None of the cells used were contaminated with mycoplasma.

The 21 pairs of KIRC and paraneoplastic tissue specimens used in this study were obtained from surgically resected patients at the First Affiliated Hospital of Soochow University. Informed consent was obtained from the patients, and the ethics committee of Soochow University approved the use of the specimens. Patients had not received radiotherapy or immunotherapy prior to specimen collection, and specimens were divided after removal and stored frozen at -80°C or in formalin at room temperature until further processing.

### 2.10 Western blot assessment

Cells were hydrolyzed in RIPA buffer containing a mixture of protease inhibitors and phosphatase inhibitors. Protein products were separated by SDS-PAGE, transferred to nitrocellulose membranes, and cut according to the molecular weight of the target proteins. The membranes were then blocked in TBST buffer containing 3.0% BSA for 1 hour and incubated with primary antibody at 4°C overnight. The membranes were then rinsed three times for 10 minutes each with TBST buffer and incubated with the appropriate secondary antibody for 2 hours at room temperature. Protein expression was detected using an enhanced chemiluminescence system. The expression of the target protein was normalized to the expression of GAPDH or tubulin. The primary antibodies used for WB analysis were as follows: anti-mouse E-cadherin antibody, anti-mouse N-cadherin antibody (BD Biosciences, USA); anti-mouse snail antibody (Cell Signaling Technology, USA); anti-mouse vimentin antibody (Santa Cruz, USA); anti-rabbit BCAT1 antibody, anti-mouse GAPDH antibody, anti-mouse tubulin antibody (Abclonal, China). The secondary antibodies were: goat anti-mouse IgG antibody, goat anti-rabbit IgG antibody (Abclonal, China). The relative expression of proteins was normalized to the expression of the same group of lysate samples were run in parallel gels to test different proteins.

### 2.11 RNA extraction and qRT-PCR assays

Total RNA was extracted using the TRIzol RNA protocol, and cDNA was synthesized by reverse transcription using the HiScript^®^ III RT SuperMix for qPCR kit (Vazyme, China). BCAT1 mRNA expression was detected by qRT-PCR and normalized to β-actin. The target gene expression levels were calculated relative to β-actin. Finally, the expression level was quantified using the ΔΔCt method (54). The primers used in the experiment included: BCAT1 (Forward primer: GTGGAGTGGTCCTCAGAGTTT; Reverse primer: AGCCAGGGTGCAATGACAG); β-actin (Forward primer. CATGTACGTTGCTATCCAGGC; Reverse primer: CTCCTTAATGTCACGCACGAT).

### 2.12 RNA interference and overexpression plasmid

The sequences of siRNAs(GenePharma, China) were as follows: si-BCAT1-1(5’-CCCAAUGUGAAGCAGUAGAUA-3’) and si-BCAT1-2(5’-CCUGUGUUGUUUGCCCAGUUU-3’). The shRNAs used the same sequence as the siRNAs and were synthesized into the pLKO3.1 plasmids (Tsingke Biotechnology, China). The overexpression plasmid pCDH-BCAT1-3xFlag (Tsingke Biotechnology, China) was synthesized based on the coding sequence of BCAT1 (NM_005504.7). 786-o and 769-p cells were transfected with Lipofectamine 3000 (Invitrogen, USA) using the aforementioned siRNA and plasmids.

### 2.13 Wound-healing (cell migration) assay

The 786-o and 769-p cells were evenly spread on the well plates, and when the cells reached 95% density, the cells were gently scraped along a straight line with the tip of a sterilized pipette, with 5 lines through each well, each line kept parallel and equally spaced. Serum-free medium was added and incubated in a 37°C, 5% CO_2_ incubator for 12 hours (55). Microscopic photographs of the cells were taken at five randomly scratched areas, and then the distance of cell migration to the scratched areas was analyzed using ImageJ software. The experiment was repeated three times.

### 2.14 Transwell(cell migration and invasion) assays

Transwell chambers (BD Biosciences, USA) were placed in 24-well culture plates. The surface of the chambers used in the invasion assay must be covered with a layer of matrix gel. In each upper chamber, 786-o and 769-p cells were added with 1% FBS medium, and 20% FBS medium was placed in the lower chamber as a chemoattractant. The 24-well plates were incubated in a 37°C, 5% CO_2_ incubator for 24 hours, and the upper surfaces of the chambers were cleaned with a swab. The cells in the bottom layer were stained with 1% crystal violet stain, three microscopic images were taken, and the cells were counted. Results were tested in triplicate.

### 2.15 Immunohistochemistry

Paraffin-embedded tumors and paracancerous tissues were sectioned for IHC assays (15), and the following antibodies were used: anti-rabbit BCAT1 antibody (Proteintech, USA); EnVision™ FLEX+ Rabbit (LINKER) (Dako, Denmark) was chosen as the secondary antibody.

### 2.16 Statistical analysis

The analysis of all data and graphs was performed using the Sento Academic Platform software(www.xiantaozi.com) and R software (version number 3.6.3). Differences between two sample means were analyzed using the t-test, and one-way ANOVA was applied to the analysis of differences between more than two samples. We considered p values <0.05 as statistically significant(*p < 0.05, **p < 0.01,***p<0.001).

## 3 Results

### 3.1 scRNA-seq and cell annotation of KIRC samples

**Figure 1:**
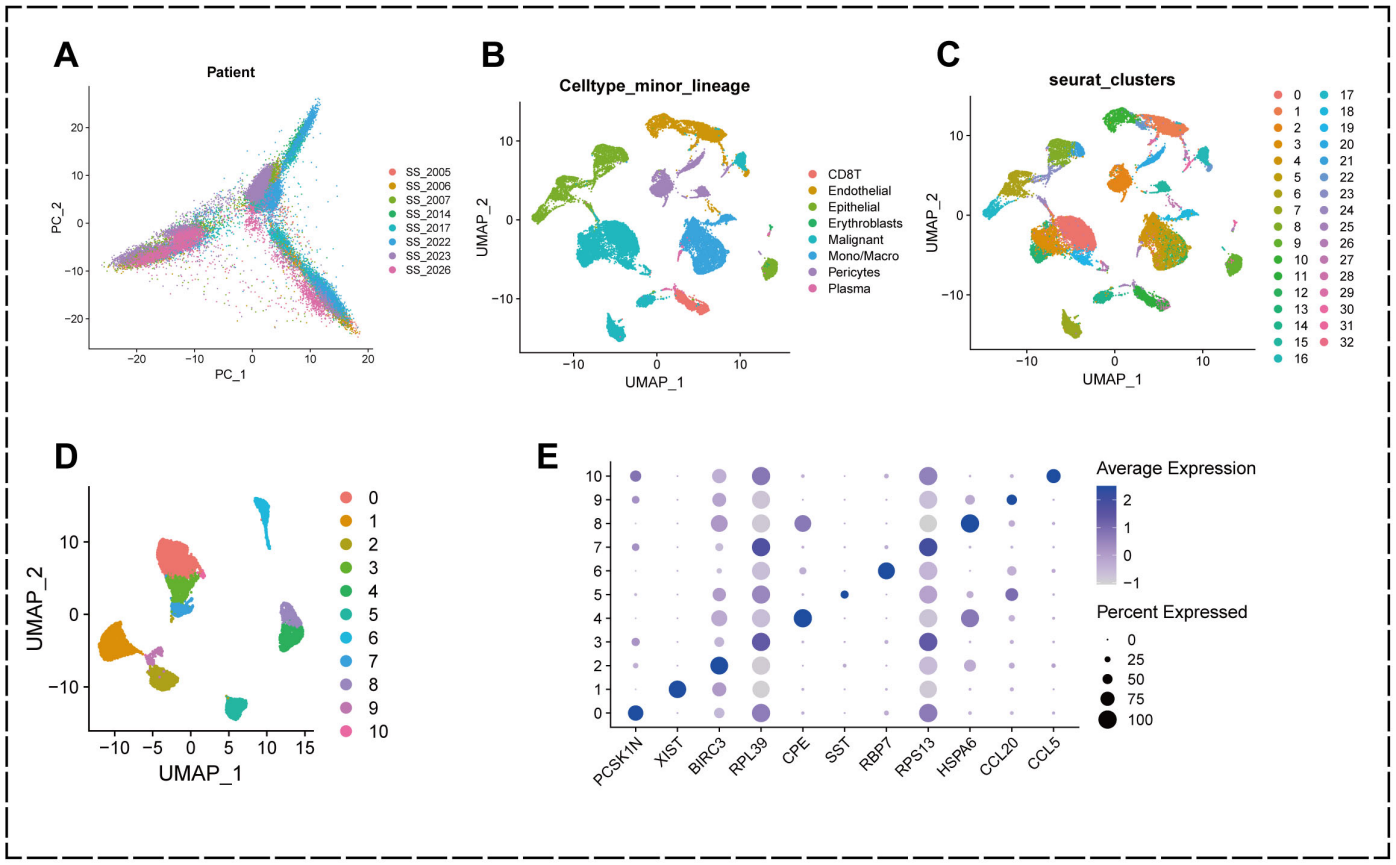
Annotation of single-cell data and extraction of malignant epithelial cells. **(A)** PCA plot showing no significant batch effect between cells from different samples. **(B)** UMAP plot showing that all cells in the 8 KIRC samples can be categorized into 33 cell clusters. **(C)** UMAP plot showing that the KIRC samples can be annotated into 8 cell types in the tumor microenvironment. **(D)** malignant cells were categorized into 11 subclusters. **(E)** Bubble diagram showing genes characterized by different subclusters.

To analyze the cellular composition of the tumor microenvironment(TME) in KIRC, the most predominant subtype in renal cell carcinoma, we extracted eight quality controlled and standardized KIRC single-cell gene expression profiles from the single-cell transcriptome sequencing dataset GSE159115 to characterize the cellular populations of human KIRC. The samples from these eight patients were quality-controlled to contain a total of 27,669 cells. To detect possible batch effects in single-cell RNA sequencing (scRNA-seq) data, we performed principal component analysis (PCA) and demonstrated that there were no significant batch effects for cells in different samples (**Figure 1A**).

We used the R package “Seurat” to perform UMAP downscaling and clustering of these cells, and classified the cells in the KIRC tumor microenvironment into 33 clusters based on the similarities and differences in their expression patterns (**Figure 1B**).

Further cell annotation results showed that the 33 cell clustering subgroups could be annotated into 8 classes of cells, namely CD8+ T cells, endothelial cells, epithelial cells, erythroblasts, malignant cells, monocytes/macrophages, pericytes, and plasma (**Figure 1C**).

To better understand the heterogeneity of malignant epithelial cells in the TME of KIRC and their potential value for prognosis and drug therapy screening, we extracted malignant epithelial cell types at the single cell level and further subdivided them into subpopulations using the “Seurat” package. The results showed that malignant epithelial cells could be classified into 11 subpopulations (0-10) based on different molecular markers (**Figures 1D, E**).

This indicates that there is heterogeneity of gene expression in different clusters of malignant epithelial cells in KIRC patients, and these findings suggest that the heterogeneity of malignant epithelial cells in the tumor microenvironment of KIRC may play a crucial role in the development of KIRC.

### 3.2 Malignant epithelial cell trajectory analysis and EMT-related gene screening

**Figure 2:**
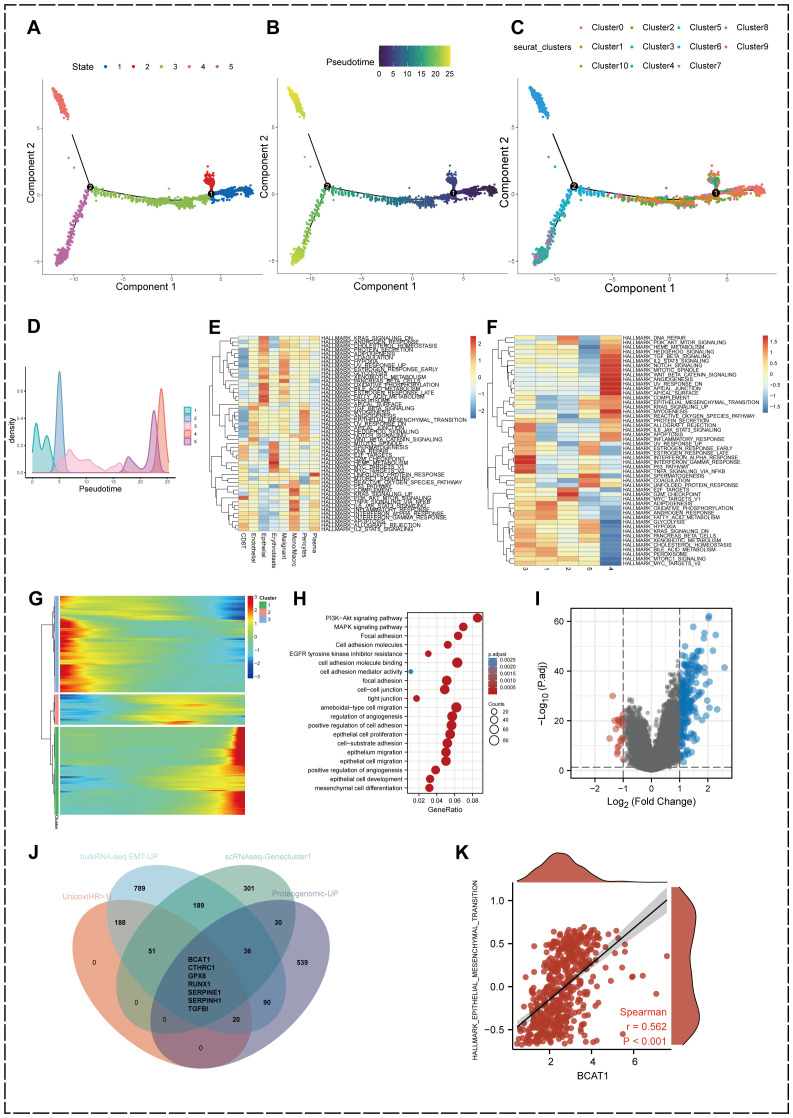
Identification of heterogeneous genes associated with differentiation trajectories of malignant cells. **(A)** Analysis of differentiation trajectories of malignant cells. **(B)** Distribution of different subclusters of malignant cells on differentiation trajectories. **(C, D)** Pseudotime analysis of malignant cells. **(E)** GSVA analysis showing the enrichment levels of eight cell types in different Hallmark pathways in KIRC samples. **(F)** GSVA analysis of the differentiation status of malignant epithelial cells. **(G)** Heatmap showing malignant cells can exhibit 3 expression patterns after differentiation. **(H)** GO enrichment analysis of heterogeneous genes associated with malignant cells differentiation. **(I)** Differentially expressed genes between high EMT and low EMT groups. (|Fold Change| > 2, p.adjust < 0.05) **(J)** Comprehensive scRNA-seq-GeneCluster1, bulkRNA-seq EMT-related upregulated genes, intersecting genes of proteogenomic upregulated proteins and prognostic conditions. **(K)** Association between BCAT1 expression levels and Hallmark-EPITHELIAL-MESENCHYMAL-TRANSITION gene set enrichment score correlation.

Gene expression heterogeneity among KIRC malignant epithelial cells may be involved in differences in the biological behavior of cancer cells, and to further identify cell subpopulations and molecular markers that are closely associated with KIRC progression, we performed an analysis of differentiation trajectories (**Figure 2A**). The results revealed that the 11 malignant epithelial cell subpopulations could be divided into five differentiation stages.

We found that different subpopulations of malignant epithelial cells were differentially distributed in differentiation trajectories (**Figure 2B**). We then performed pseudotime analysis of malignant epithelial cells (**Figures 2C, D**), the purple part is the initial state of differentiation while the yellow is the terminal state of differentiation, and it can be found that cell state 4 is located at the terminal stage of differentiation.

GSVA analysis demonstrated the activation levels of 50 Hallmark pathways associated with cancer in different cell types in the TME of KIRC (**Figure 2E**). GSVA also demonstrated the activation levels of Hallmark pathways associated with cancer in different differentiation states of malignant epithelial cells (**Figure 2F**), in which transforming growth factor-β (TGF-β), PI3k-AKT-mTOR, Wnt-β-Catenin, reactive oxygen species (ROS), angiogenesis and other important signaling pathway activation levels were significantly upregulated.

In addition, we found that the heterogeneous genes associated with the differentiation trajectory of malignant epithelial cells showed three expression patterns after clustering (**Figure 2G**), and we divided three geneclusters accordingly. genecluster1 showed a high upregulation at the terminal stage of the proposed time sequence. We performed GO enrichment analysis of gencluster1 (**Figure 2H**), which showed that these genes were significantly enriched in signaling pathways related to cell adhesion, epithelial cell migration and development, such as Focal adhesion, Cell adhesion molecules, and tight junction. In addition, signaling pathways such as phosphatidylinositol-3-kinase-protein kinase B (PI3K-Akt) and mitogen-activated protein kinase (MAPK) were also significantly activated.

We performed GSVA analysis of the HALLMARK- EPITHELIAL-MESENCHYMAL-TRANSITION gene set using KIRC mRNA expression data from the TCGA database to obtain differentially expressed genes in samples with high versus low EMT levels (**Figure 2I**). 1370 genes were upregulated in the high EMT-enriched subgroup from the bulk RNA-seq level these genes are participating in the EMT process in KIRC.

On the other hand, we introduced proteins that had been reported to be significantly upregulated in KIRC in a proteogenomic study by Clark et al (56). Genes with remarkable significance in scRNA-seq, bulk RNA-seq, proteogenomics and prognostic analysis were jointly intersected and screened for seven key genes affecting the EMT process in KIRC including BCAT1, CTHRC1, GPX8, RUNX1, SERPINE1, SERPINH1 and TGFBI (**Figure 2J**).

We traversed the literature and observed that BCAT1 is significantly upregulated during progression of various types of cancer and has emerged as an essential biomarker, but its role in KIRC is poorly understood. Correlation analysis demonstrated that BCAT1 expression was highly correlated with the HALLMARK-EPITHELIAL-MESENCHYMAL-TRANSITION gene set (r=0.562, p<0.001) (**Figure 2K**). Thus, we decided to further explore the role and mechanism of BCAT1 in KIRC metastasis and development.

### 3.3 Genetic alteration of BCAT1 gene in KIRC

**Figure 3:**
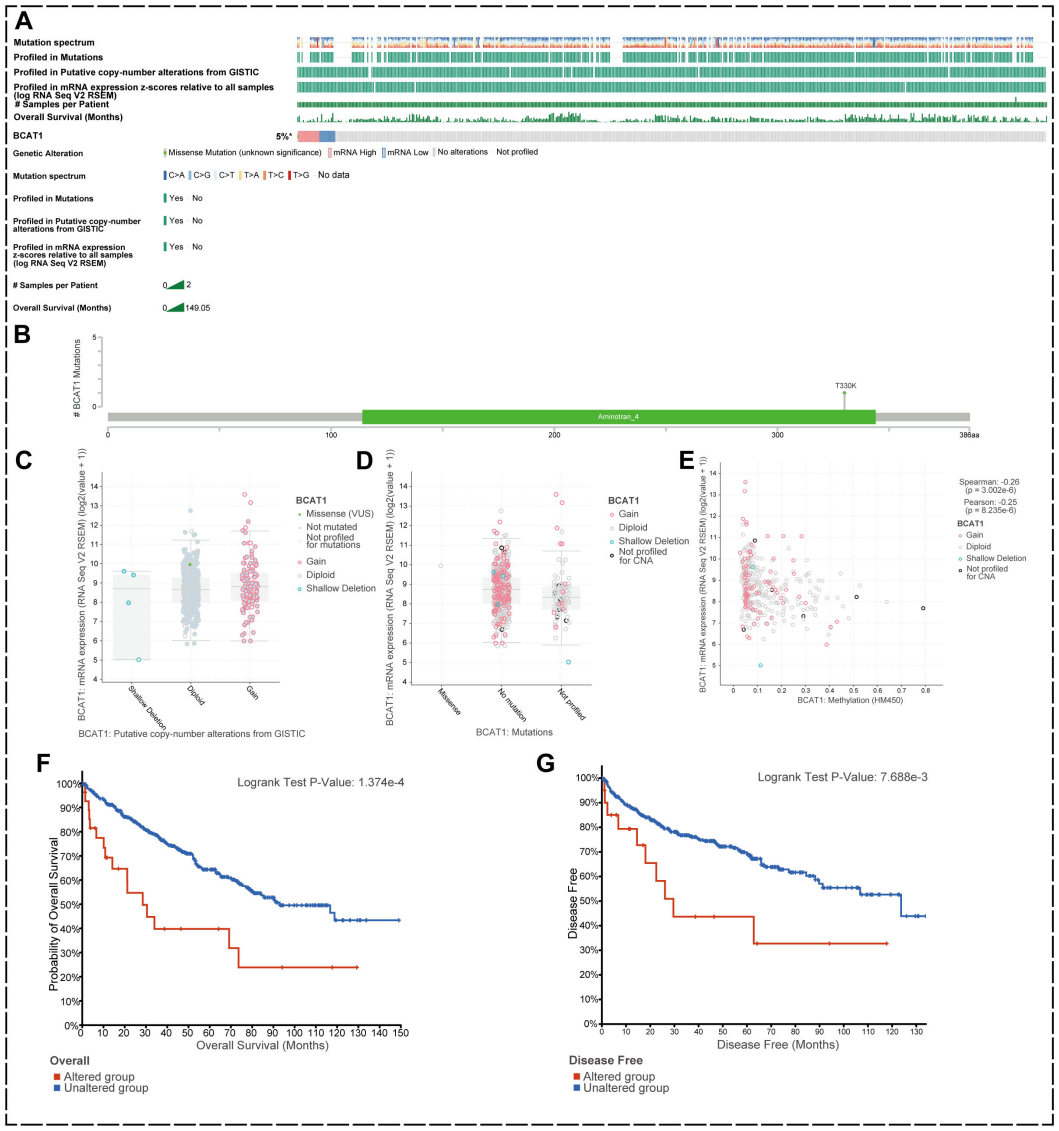
BCAT1 genetic alterations in KIRC. **(A)** Mutations in BCAT1 in 538 KIRC samples. **(B)** Somatic mutations of BCAT1. **(C, D)** BCAT1 mRNA expression levels did not differ significantly between CNV copy numbers, nor did they differ significantly between mutations. **(E)** The mRNA expression level of BCAT1 was negatively correlated with the methylation level of the promoter region of BCAT1. methylation level of the BCAT1 promoter region. **(F, G)** Correlation of prognosis between BCAT1 and mutations in KIRC.

We used the cBioPortal tool to analyze mutations in the BCAT1 gene in KIRC using the Kidney Renal Clear Cell Carcinoma (TCGA, Firehose Legacy) dataset, which contains 538 KIRC samples (**Figure 3A**).

We found that the overall genetic variation rate of BCAT1 in KIRC was 5%, with alterations at the mRNA level being the predominant form (**Figure 3B**). There was one somatic mutation site in the amino acid sequence of BCAT1 where missense mutations occurred. Analysis revealed no significant differences in BCAT1 mRNA expression levels between CNV copy number amplification and deletion, nor between mutation and non-mutation (**Figures 3C, D**). However, we found that methylation of the promoter region of BCAT1 was negatively correlated with BCAT1 mRNA expression (r=-0.17, p<0.05) (**Figure 3E**).

We further investigated the effect of BCAT1 gene mutation on patient prognosis. The results showed that overall survival (OS) and disease-free survival (DFS) were significantly decreased in the mutated group compared with the non-mutated group of KIRC patients (**Figures 3F, G**). Taken together, these data suggest that mutations in BCAT1 may have an impact on tumor progression and thus patient prognosis.

### 3.4 Expression levels of BCAT1 in KIRC and prognosis of patients

**Figure 4:**
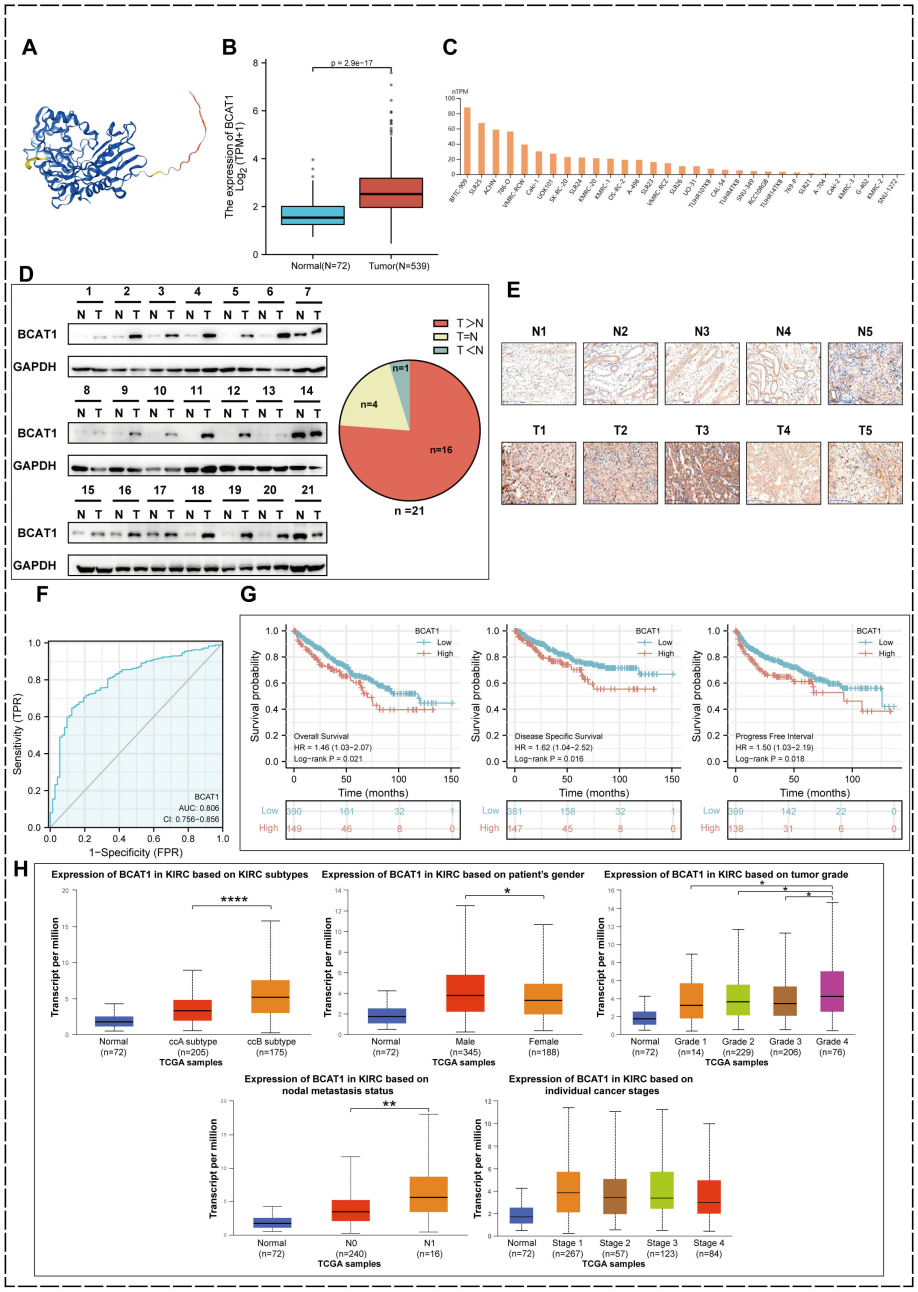
BCAT1 expression in KIRC and prognostic value. **(A)** The protein encoded by BCAT1. **(B)** Relative expression levels of BCAT1 between KIRC and paracancerous tissues in the TCGA database. **(C)** Expression levels of BCAT1 in different cell lines. **(D)** Western Blotting to detect the protein expression level of BCAT1 in clinical specimens and paracancerous tissues. (Uncropped images of blots are presented in **Supplementary File 1**) **(E)** Immunohistochemical staining to detect the protein expression of BCAT1 (Five pairs of results are shown herewith and the rest are shown in **Supplementary File 2**). **(F)** ROC curve of BCAT1 in KIRC. **(G)** Kaplan-Meier survival curves showing the effect of BCAT1 on overall survival (OS), disease-specific survival (DSS), and progression-free interval (PFI) in KIRC patients. **(H)** Expression of BCAT1 in different molecular subtypes, gender, tumor grade, N-stage, and tumor stage in the TCGA database.

We obtained the structure of the protein encoded by BCAT1 gene from The Human Protein Atlas database (**Figure 4A**). We identified BCAT1 expression levels in KIRC tumor tissues and paraneoplastic tissues in the TCGA database and found that BCAT1 expression was significantly elevated in tumor tissues (**Figure 4B**). We also found elevated levels of BCAT1 expression in multiple KIRC cell lines through the cBioPortal tool (**Figure 4C**).

We have performed Western Blot on tumor tissue and paracancerous tissue from 21 pairs of clinical specimens (**Figure 4D**) and immunohistochemical staining on 20 of them (**Figure 4E**), all of which showed significant overexpression of BCAT1 in KIRC. This validated the results in the database.

The ROC curve we plotted as well revealed that BCAT1 was used for single gene diagnosis of KIRC with high accuracy (AUC=0.806, 95% CI: 0.756-0.856) (**Figure 4F**).

We used the Kaplan-Meier method to analyze the prognostic value of BCAT1. The results (**Figure 4G**) showed that among KIRC patients, the BCAT1 high expression group had a shorter progression free interval (PFI) compared to patients in the low expression group, and OS and disease-specific survival (DSS) were similarly significantly lower.

By performing analysis through the UALCAN database, we obtained the expression of BCAT1 in different molecular subtypes, different genders, different grades, different N-stages and different tumor stages of KIRC. The results (**Figure 4H**) showed that among the different molecular subtypes, it was significantly more expressed in the ccB molecular subtype than in the ccA subtype. And the expression was higher in male patients than in females. Among the tumor grades, the highest BCAT1 expression was found in KIRC of Grade 4. BCAT1 expression was higher in patients with lymph node metastasis. There was a slight difference in BCAT1 expression in patients with different stages of KIRC.

### 3.5 BCAT1 co-expression network construction and enrichment analysis by GSEA

**Figure 5:**
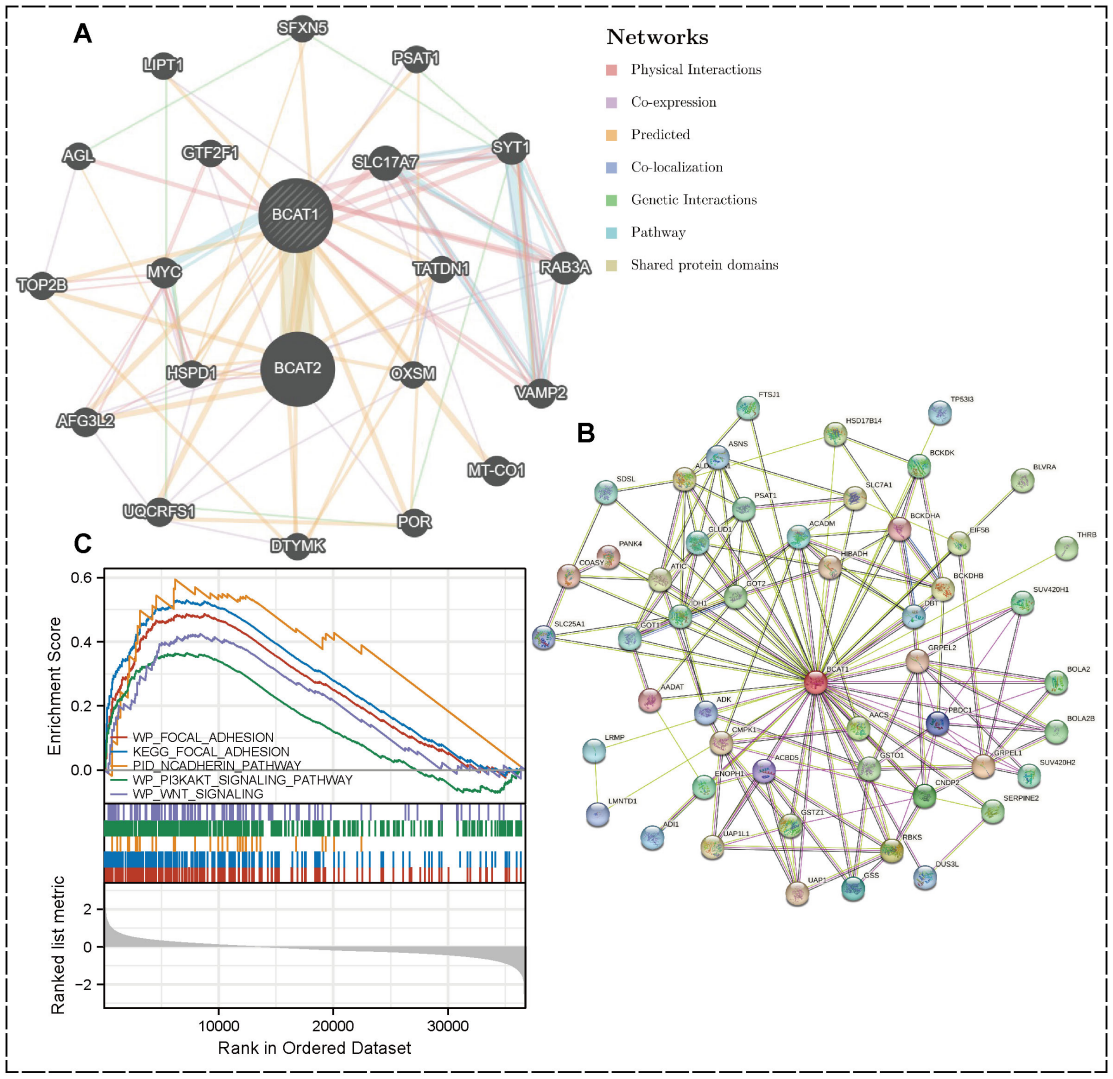
PPI network and GSEA enrichment analysis. **(A)** PPI network of BCAT1 (top 20) created by GeneMania. **(B)** Visualization by STRING showing the top 50 interacting genes. **(C)** GSEA results showing the pathway of BCAT1 enrichment in KIRC.

We constructed a protein-protein interaction (PPI) network to find BCAT1 co-expressed or interacting genes. Using the GeneMANIA database, we identified the top 20 interacting genes (**Figure 5A**). And in the STRING database, we visualized the top 50 interacting genes (**Figure 5B**). The PPI network covers seven types of linkage types: physical interactions, co-expression, predicted, co-localization, genetic interactions, pathway, and shared protein domains. it stands to reason that BCAT2 is closely related to BCAT1, and these genes may play a joint role with BCAT1 in the development of KIRC.

Enrichment analysis was performed by GSEA software, and cell adhesion-related pathways such as Focal Adhesion, N-cadherin, PI3K-AKT and WNT signaling pathways were enriched in KIRC. All these pathways are closely associated with EMT. Further suggesting that we BCAT1 may be involved in the regulation of EMT in KIRC thereby affecting its metastasis (**Figure 5C**).

### 3.6 Overexpression of BCAT1 promotes EMT in KIRC and facilitates cell migration and invasion

**Figure 6:**
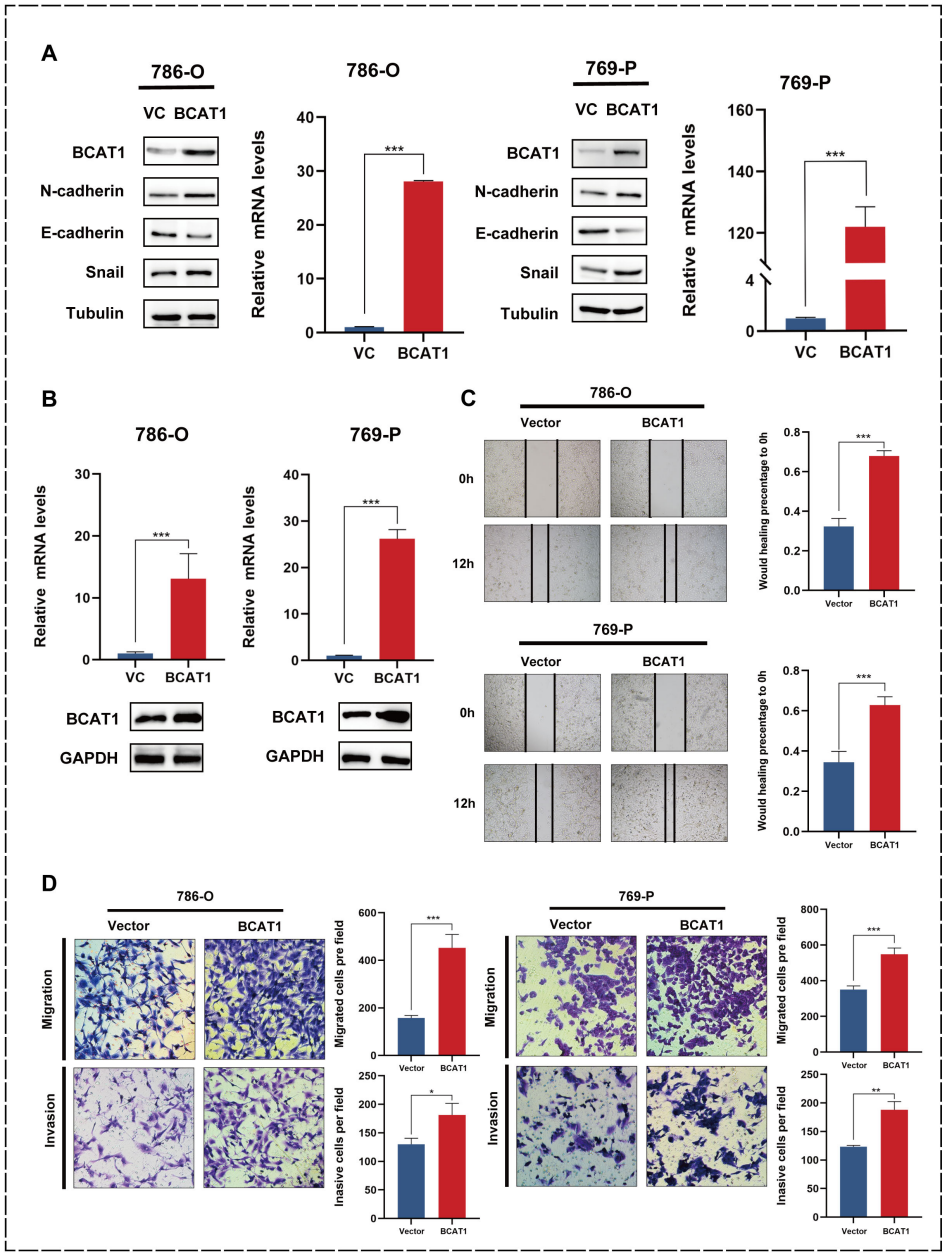
Overexpression of BCAT1 promotes EMT in 786-o and 769-p cells. **(A)** After transient overexpression of BCAT1 in 786-o and 769-p cells, the expression of BCAT1 and EMT-related marker proteins were detected by Western Blotting, and the mRNA expression of BCAT1 was detected by qPCR. (Uncropped images of blots are presented in **Supplementary File 1**) **(B)** After stable overexpression of BCAT1, the overexpression effect was detected by qPCR and Western Blotting. (Uncropped images of blots are presented in **Supplementary File 1**) **(C)** Scratch healing assay was performed on 786-o and 769-p cells stably overexpressing BCAT1, showing enhanced cell migration. **(D)** Transwell assay on 786-o and 769-p cells stably overexpressing BCAT1 showed enhanced cell migration and invasion ability.

We transiently transfected BCAT1 overexpression into 786-o and 769-p cells, and then examined the mRNA and protein expression levels of BCAT1 by qRT-PCR and Western blot. As shown in **Figure 6A**, we detected the expression of EMT-related markers by Western blot, and the expression levels of N-cadherin and Snail were significantly increased, and the expression levels of E-cadherin were significantly decreased.

We then performed stable BCAT1 overexpression in 786-o and 769-p cell lines using pCDH-BCAT1-3x Flag. We similarly validated the overexpression effect (**Figure 6B**).

The 786-o and 769-p cells overexpressing BCAT1 had enhanced migratory ability (**Figure 6C**), and the Transwell assays showed that these cells had significantly enhanced migratory and invasive abilities (**Figure 6D**).

### 3.7 Knockdown of BCAT1 inhibits EMT in KIRC and suppresses cell migration and invasion

**Figure 7:**
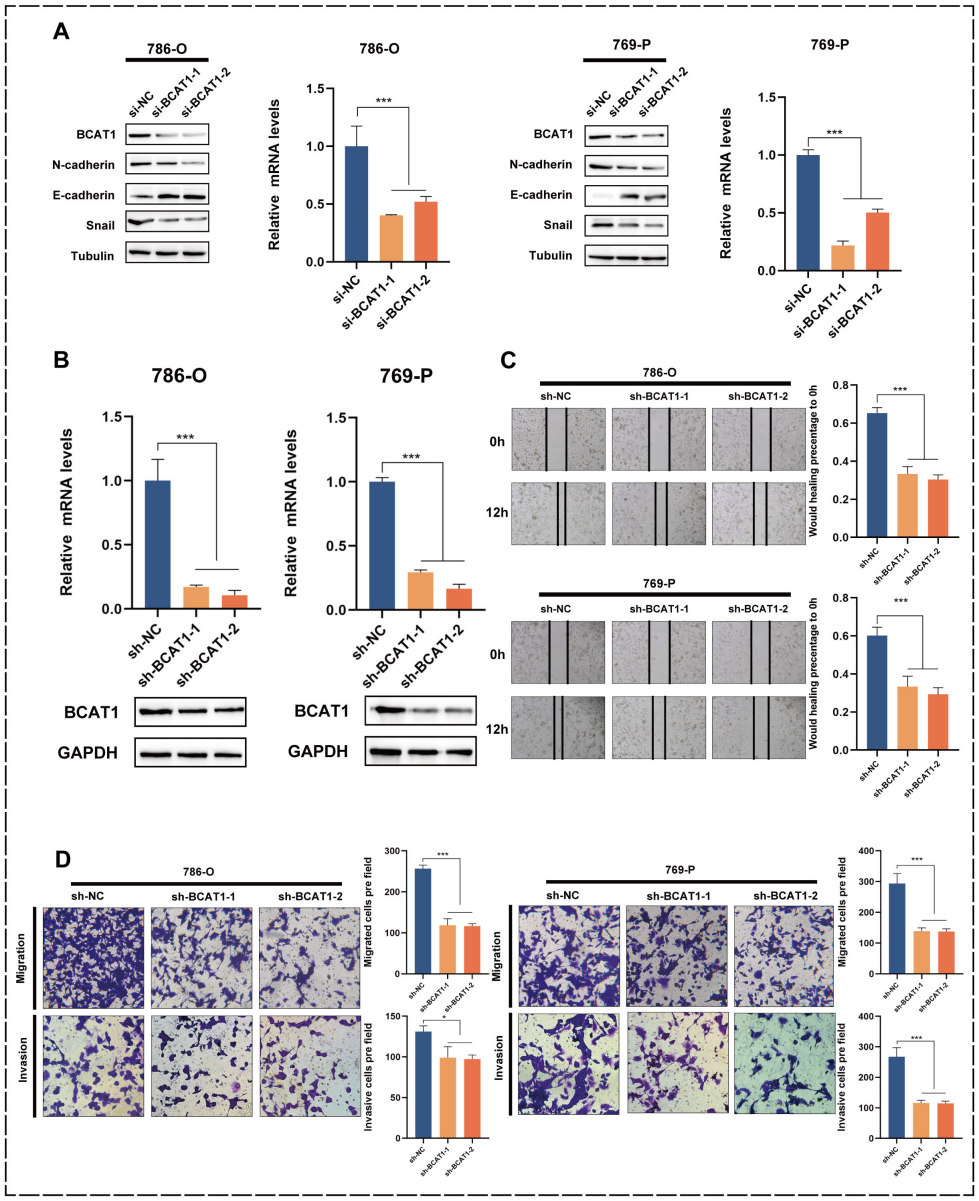
Knockdown of BCAT1 inhibits EMT in 786-o and 769-p cells. **(A)** BCAT1 was transiently knocked down in 786-o and 769-p cells, and the expression of BCAT1 and EMT-related marker proteins was detected by Western Blotting, and the mRNA expression of BCAT1 was detected by qPCR. (Uncropped images of blots are presented in **Supplementary File 1**) **(B)** After stable knockdown of BCAT1, the knockdown effect was detected by qPCR and Western Blotting. (Uncropped images of blots are presented in **Supplementary File 1**) **(C)** Scratch assay showed that the migration ability of 786-o and 769-p cells with stable knockdown of BCAT1 was weakened. **(D)** Transwell assay showed that 786-o and 769-p cells with stable knockdown of BCAT1 had decreased migration and invasion ability.

In addition, we transiently knocked down BCAT1 in 786-o and 769-p using siRNA. We verified the effect at the mRNA level using qRT-PCR and at the protein level using Western blot (**Figure 7A**). After BCAT1 was successfully knocked down, the protein expression levels of EMT-related markers changed, with a significant decrease in N-cadherin and Snail expression levels and a significant increase in E-cadherin expression levels. After constructing BCAT1 stably knocked-down cells by the above method (**Figure 7B**), scratch healing assays showed a significant decrease in the migratory ability of these cells (**Figure 7C**). Transwell assays also showed that BCAT1 knockdown significantly inhibited the migratory and invasive ability of the cells (**Figure 7D**).

### 3.8 Correlation of BCAT1 with immune cells

**Figure 8:**
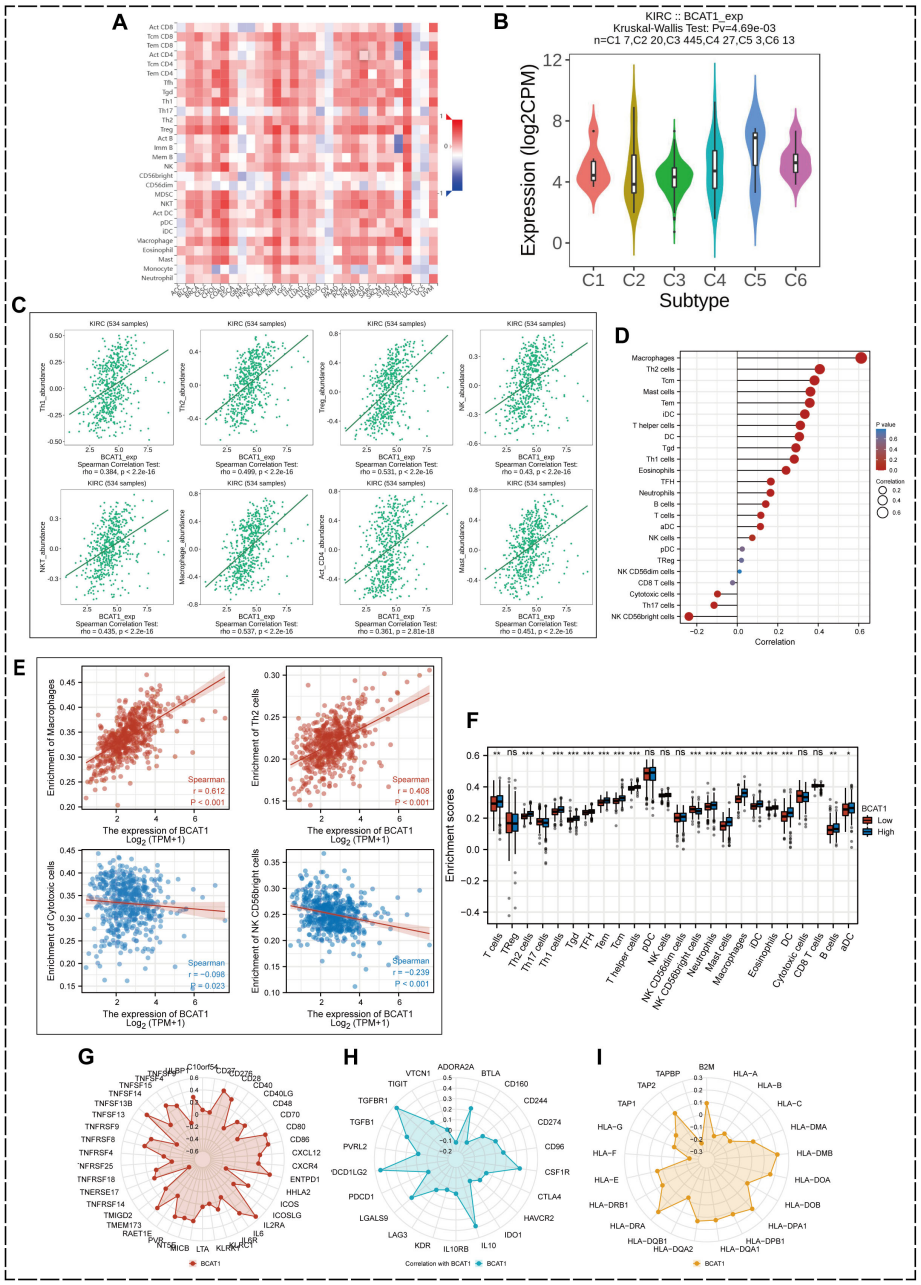
Correlation between BCAT1 and immune cells. **(A)** Correlation between BCAT1 expression and immune cell infiltration. **(B)** Relative expression of BCAT1 between different KIRC immune subtypes. **(C)** Positive correlation between the infiltration abundance of multiple immune cells in KIRC and BCAT1 expression. **(D, E)** ssGSEA showing the correlation between BCAT1 reach and common immune cell enrichment in KIRC. **(F)** Differences in immune cell infiltration between groups with high and low levels of BCAT1 mRNA expression. **(G–I)** Correlation of immune activation checkpoints, immune suppression checkpoints and MHC molecules with BCAT1 expression.

Using the TISIDB database, we investigated the correlation between BCAT1 expression and various immune cell infiltrations based on pan-cancer samples (**Figure 8A**), and found that BCAT1 was closely associated with various immune cell infiltrations in most cancers. BCAT1 was differentially expressed in different immune subtypes of KIRC, with the highest expression in the c5 immune subtype (**Figure 8B**). In 534 KIRC samples, the infiltration abundance of most immune-associated cells showed a positive correlation with the mRNA expression level of BCAT1 (**Figure 8C**), including Th1 cells, Th2 cells, regulatory T cells (Treg), natural killer (NK) cells, and macrophages. These results suggest that BCAT1 expression may be involved in the regulation of immune infiltration in the KIRC tumor microenvironment.

To further determine the relationship between BCAT1 expression and immune cell infiltration in KIRC, we used the ssGSEA algorithm to enrich the gene set of 24 characteristic immune cell markers (**Figure 8D**) and analyzed the correlation between BCAT1 expression and the degree of enrichment of common immune cells in KIRC, in which macrophages and Th2 cells were significantly enriched with increased BCAT1 expression enrichment, while the opposite was true for cytotoxic cells and the CD56bright subpopulation of NK cells (**Figure 8E**).

We also compared the differences in immune cell infiltration between the high and low BCAT1 mRNA expression groups, which showed significant differences in macrophages, Th2 cells, Th1 cells, T cells, and the CD56bright subpopulation of NK cells (**Figure 8F**). In addition, radargrams visualized the correlation of immune activation checkpoints, immune suppression checkpoints and major histocompatibility complex (MHC) molecules with BCAT1 expression levels (**Figures 8G–I**).

### 3.9 Univariate analysis of the prognostic value of BCAT1 and the prognostic model

**Figure 9:**
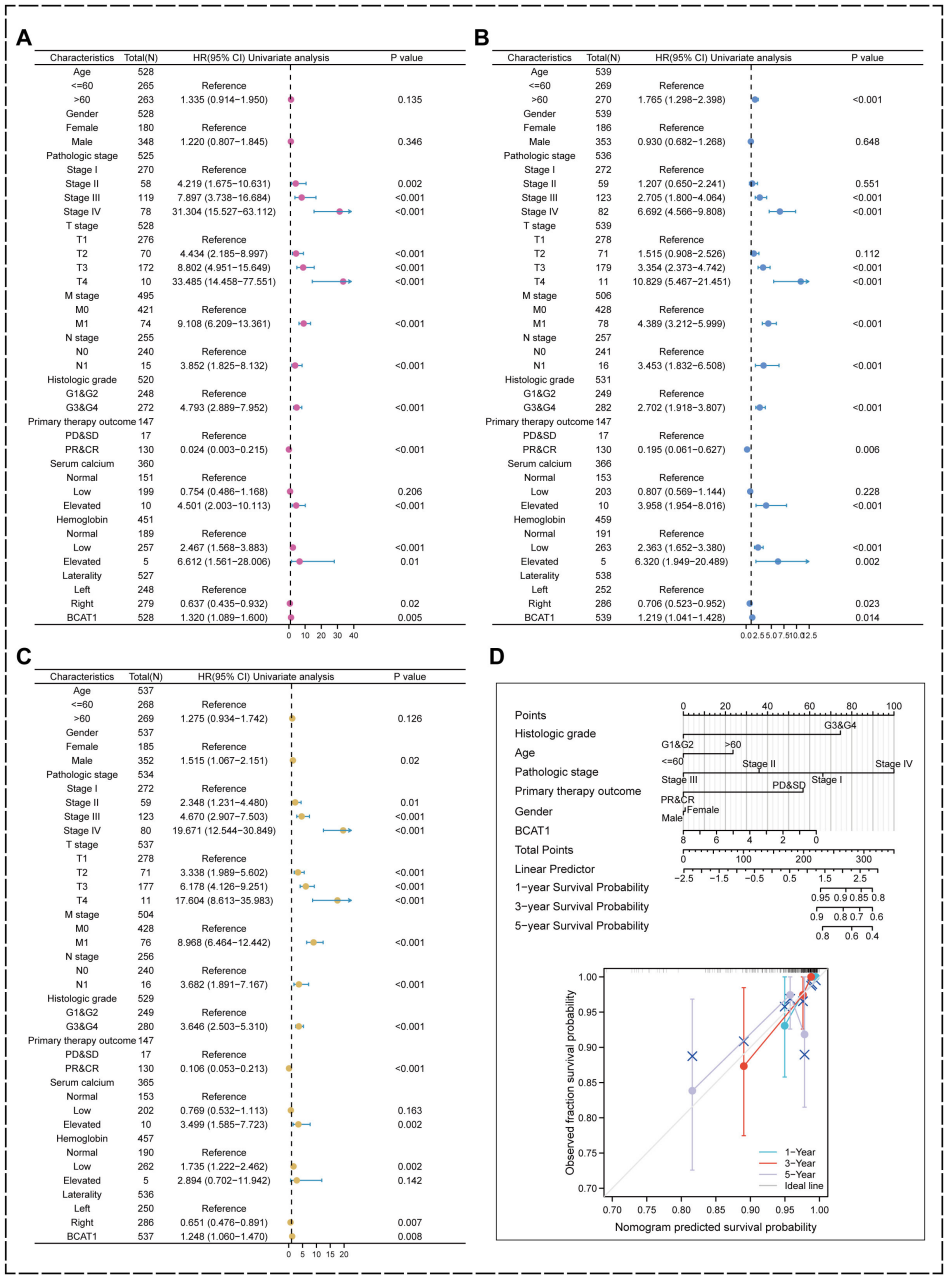
Univariate analysis and prognostic modeling of the prognostic value of BCAT1. **(A–C)** BCAT1 possesses significant prognostic predictive value in DSS, OS, and PFI indicators in KIRC. **(D)** Nomogram was used to predict the prognosis of KIRC patients.

We performed univariate analysis on the KIRC samples from the TCGA database. The results showed that BCAT1 had significant prognostic predictive significance on DSS, OS and PFI, as well as pathological stage, TMN stage, pathological grading, immunotherapy outcome and serum calcium (**Figures 9A–C**).

We also included pathological grading, age, pathological staging, immunotherapy outcome, and gender combined with BCAT1 mRNA expression levels to establish a risk prognosis model using multifactorial regression analysis and plotted a nomogram, and the higher the score, the worse the prognosis as measured by the nomogram. The calibration curve showed that the nomogram has good performance in predicting prognosis (**Figure 9D**). It can be used to comprehensively predict the prognosis of KIRC patients.

### 3.10 Correlation of BCAT1 promoter region methylation with KIRC clinical factors and BCAT1 mRNA level expression

**Figure 10:**
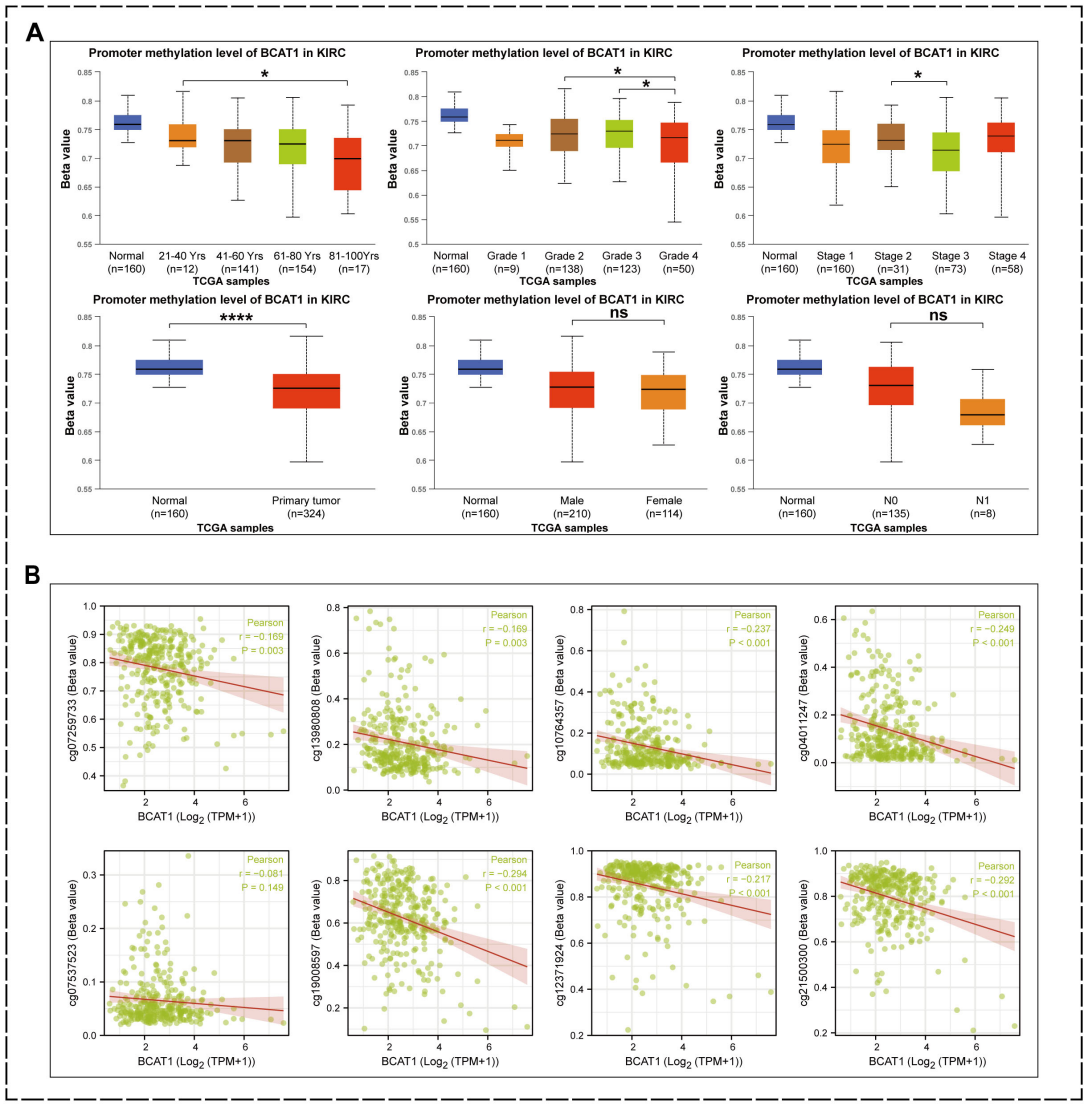
Factors associated with low methylation levels in the BCAT1 promoter region. **(A)** Reduced methylation levels of BCAT1 promoter regions were associated with high mRNA expression of BCAT1, advanced age of patients, higher Grade, and later Stage. **(B)** BCAT1 mRNA levels were negatively correlated with methylation levels in these promoter regions.

As mentioned above, the methylation level of BCAT1 promoter region was significantly and negatively correlated with the mRNA expression of BCAT1, and hypomethylation of BCAT1 promoter region might promote the expression of BCAT1. Therefore, we further analyzed the correlation between the methylation levels of BCAT1 promoter region and clinical factors by UALCAN (**Figure 10A**). The analysis showed significant differences in the methylation level of this promoter region between different ages, grades and stages, while there were no significant differences between different genders and different N stages. Overall, the methylation level of BCAT1 promoter region was significantly lower in KIRC tumor tissues than in normal kidney tissues. Subsequently, our further analysis showed that the promoter region methylation levels of cg07259733, cg21500300, cg12371924, cg19008597, cg07537523, cg04011247, cg10764357, and cg13980808 were significantly negatively correlated with BCAT1 mRNA expression (**Figure 10B**).

## 4 Discussion

Kidney cancer has the highest mortality rate of the three major urologic tumors. Renal cell carcinoma accounts for more than 90% of renal cancer cases. The mortality rate of renal cell carcinoma is slowly decreasing worldwide, benefiting from the development of treatment modalities and increased frequency of medical screening (40). KIRC, the predominant type of renal cell carcinoma, has a well-established immunotherapy system (57, 58), but the search for new targets or valuable biomarkers is of great value for both the treatment and prognosis of KIRC.

In the present study, we first profiled KIRC at the single-cell level using publicly available bioinformatics data. Based on information from the GSE159155 database, heterogeneity in the differentiation trajectory of KIRC malignant epithelial cells was revealed, with high activation of key signaling pathways such as EMT, TGF-β, Notch, IL2-STAT5, WNT-β-catenin, ROS, and angiogenesis in cells at the end of the differentiation trajectory. GO analysis revealed that high expression of terminal differentiation genes was enriched in EMT and cell adhesion-related pathways such as PI3K-AKT and MAPK. Combining prognostic information, proteogenomic data, bulk rna-seq and scRNA-seq data, we screened the target gene of interest, BCAT1.

BCAT1, as a member of the branched-chain amino acid transferases, is an important component of amino acid metabolism. BCAT1 is highly expressed in a variety of cancers, promotes cancer cell migration and invasion, and has received much attention in recent years.

BCAT1 has been reported to be highly expressed in a variety of cancers and to be associated with poor progression and prognosis. Renal cancer has seldom been linked to BCAT1. Only one researcher found that BCAT1 was co-expressed in hypertension and renal cell carcinoma by bioinformatic analysis and may be a key gene in hypertension-associated renal cell carcinoma (59).

The pro-carcinogenic mechanism of BCAT1 varies in different tumors. BCAT1 may drive the ammonification of BCAA in the circulation, causing these cancer cells to accumulate BCAA (8), bind to Sestrin2, activate the mTORC1 signaling pathway, phosphorylate downstream effector molecules, and regulate important cellular biological processes (60). It has been reported that EZH2 inactivation and oncogenic NRAS mutations together activate BCAT1, enhance BCAA metabolism and mTOR signaling, and promote the development of myeloid leukemia (9). Luo et al. also reported that BCAT1 induces mTOR-mediated autophagy and reduces the sensitivity of hepatocellular carcinoma cells to cisplatin (15). In gastric cancer, BCAT1 acts as an oncogene by activating the PI3K/AKT/mTOR signaling pathway to promote proliferation, invasion, and angiogenesis (61). BCAT1 activates the mTORC1 pathway to play a pro-oncogenic role in breast and endometrial cancers, while BCAT1 inhibits mitochondrial reactive oxygen species (ROS) production in breast cancer cells (25, 27, 32).

In the present study, we found that BCAT1 was enriched in pathways such as PI3K/AKT and WNT in KIRC by GSEA pathway enrichment analysis. Several reports have shown that EMT can be regulated by regulating PI3K/AKT/mTOR or WNT signaling pathways (62–66), thereby affecting tumor invasion and metastasis.

Increased BCAA levels promote tumor growth, while increased BCAA catabolism also promotes tumor growth. In glioma cells, BCAA degradation by BCAT1 and increased glutamate formation provide the necessary nitrogen source for the synthesis of some non-essential amino acids (aspartate, serine, and alanine) as well as nucleotides, leading to increased proliferation, migration, and invasiveness of tumor cells *in vitro* (12). In contrast, in acute myeloid leukemia, glioma, and lung cancer, massive expression of BCAT1 reduces the level of its substrate α-KG and promotes tumor progression (4, 11, 12, 36). This may be due to the fact that a significant decrease in a-KG affects the activity of α-KG-dependent dioxygenases, which play an important role in hypoxic response and epigenetics.

Epigenetically, the pattern of methylation that occurs in the promoter region of BCAT1 is highly correlated with disease onset and progression. In colorectal cancer, researchers found significantly higher levels of BCAT1 methylation in circulating tumor DNA (ctDNA) than in normal controls (19, 20, 67–70). In a related study in ovarian cancer, BCAT1 was significantly hypermethylated in low malignant potential (LMP) and high grade (HG) plasmacytoid epithelial ovarian tumors (31). And investigators studying adverse outcomes in non-alcoholic fatty liver disease showed that high BCAT1 expression and hypomethylation predicted an increased incidence of adverse outcomes such as hepatocellular carcinoma (HCC) (71). Other investigators have reported increased BCAT1 expression and decreased BCAT1 promoter methylation levels in most hepatocellular carcinomas (72).

In our study, the results of cBioPortal analysis showed that the methylation level of the promoter region of BCAT1 was significantly and negatively correlated with the mRNA expression of BCAT1. And by using the UALCAN tool, we found that the methylation level of BCAT1 was decreased in KIRC tumor tissues. Hypomethylation of BCAT1 was also highly correlated with factors such as tumor progression and advanced age in various clinical features.

The cBioPortal analysis showed that BCAT1 gene mutation caused a significant decrease in OS and DFS in KIRC patients, making the prognosis worse. In contrast, among KIRC patients in the TCGA database, we plotted Kaplan-Meier curves showing shorter OS, PFI and DSS in the BCAT1 high-expression group compared with the low-expression group. ROC curves showed excellent prognostic predictive value of BCAT1. Elevated BCAT1 expression is associated with poor prognosis in KIRC patients, as evidenced by shorter OS, PFI and DSS. As a prognostic biomarker, BCAT1 could be used to stratify patients based on risk and guide personalized treatment strategies. Additionally, targeting BCAT1 may offer new therapeutic avenues for combating KIRC progression and metastasis.

In our analysis of TCGA data using UALCAN, we found that BCAT1 was highly expressed in the tumor tissues of KIRC patients and that patients with high BCAT1 expression had higher tumor grades and more lymph node metastases. BCAT1 expression also differed between sexes and molecular subtypes. cBioPortal also showed that BCAT1 expression was upregulated in KIRC cell lines. We collected clinical tissue samples and verified the high expression of BCAT1 in KIRC by Western blot and immunohistochemical staining.

Then, we verified the relationship between BCAT1 and EMT pathway in KIRC by *in vitro* experiments. EMT was promoted in KIRC cells after BCAT1 overexpression, and both migration and invasion abilities were significantly enhanced. EMT was inhibited in KIRC cells after BCAT1 silencing, and migration and invasion abilities were decreased.

We constructed the PPI network. The 20 and 50 genes that most significantly interacted with BCAT1 were identified using GeneMANIA and STRING databases, respectively.

In this study, we also analyzed the role of BCAT1 in the immune microenvironment. High BCAT1 expression was highly correlated with the activation of several immune-related cells in tumors. The immune system became more active in BCAT1 mutant cancers. We also found that BCAT1 expression was strongly associated with increased abundance of Treg cells, Th2 cells, and other cells. ssGSEA analysis showed the correlation between high BCAT1 expression in KIRC and the degree of enrichment of common immune cells, which was positively correlated with the enrichment of macrophages, Treg, and Th2 cells, while cytotoxic cells and the CD56bright subpopulation of NK cells were negatively correlated.

Treg cells have been reported to suppress aberrant immune responses against autoantigens and anti-tumor immune responses. Large infiltration of Treg cells in tumor tissue is usually associated with poor prognosis (73). There is a drift in the Th1/Th2 balance in a variety of cancers, often to a Th2-dominant state, which may be associated with immune escape (74). M2-type macrophages have also been reported to be involved in immune escape of tumor cells (75). In contrast, NK cells and cytotoxic cells are thought to kill cancer cells (76, 77).

BCAT1 has been shown to enhance EMT by activating key signaling pathways such as PI3K/AKT and WNT (35, 61). The PI3K/AKT pathway plays a crucial role in cell proliferation, survival, and migration. Overexpression of BCAT1 activates this pathway, leading to upregulation of N-cadherin and Snail, and downregulation of E-cadherin, thus promoting the EMT process. Our study confirms this through Western Blot experiments. The WNT pathway is another key pathway regulating EMT. BCAT1 likely modulates components of the WNT signaling pathway, further promoting tumor cell invasion and migration. Our Gene Set Enrichment Analysis (GSEA) shows significant enrichment of BCAT1 in EMT-related gene sets, supporting its critical role in the EMT process.

While we identified the involvement of PI3K/AKT and WNT pathways in BCAT1-mediated EMT, the specific molecular mechanisms remain to be elucidated. Future studies should focus on identifying the direct and indirect targets of BCAT1 within these pathways and elucidating their roles in EMT and tumor progression.

Besides PI3K/AKT and WNT pathways, BCAT1 may also interact with other signaling pathways such as TGF-β, MAPK, and Notch pathways. Investigating these interactions will provide a comprehensive understanding of BCAT1’s role in KIRC.

While our *in vitro* experiments provide strong evidence, *in vivo* studies are crucial for validating the role of BCAT1 in KIRC. Future research should include animal models to confirm the efficacy and safety of targeting BCAT1 as a therapeutic strategy.

Developing BCAT1-targeting therapies holds promise not only for treating KIRC but also for other cancers where BCAT1 plays a critical role. Targeting BCAT1 in KIRC could be achieved through small molecule inhibitors, monoclonal antibodies, or RNA interference technologies. Preclinical studies are needed to evaluate the efficacy of these strategies in reducing tumor growth and metastasis. Combining BCAT1 inhibitors with existing therapies may enhance treatment outcomes for KIRC patients. Future research should focus on the preclinical validation of these therapeutic strategies in relevant KIRC models. Successful preclinical studies should lead to the initiation of clinical trials to evaluate the safety, efficacy, and optimal dosing of BCAT1-targeting therapies in patients with KIRC. Additionally, understanding the molecular mechanisms underlying BCAT1’s role in KIRC progression and its interaction with other oncogenic pathways will help refine and optimize therapeutic approaches.

Our study is limited by the lack of *in vivo* validation, which is crucial for confirming the role of BCAT1 in KIRC. Future research should include animal studies and clinical trials to further explore BCAT1 as a therapeutic target. Additionally, investigating the interplay between BCAT1 and the immune microenvironment in KIRC could provide deeper insights into its role in cancer progression.

## 5 Conclusions

Our study reveals that BCAT1 is significantly overexpressed in kidney renal clear cell carcinoma (KIRC) and promotes epithelial-mesenchymal transition (EMT), enhancing the migratory and invasive capabilities of KIRC cells. High BCAT1 expression correlates with poor prognosis, including shorter overall survival (OS), progression-free interval (PFI), and disease-specific survival (DSS).

BCAT1 could serve as a prognostic biomarker and a therapeutic target. Future research should focus on *in vivo* validation and developing BCAT1-targeted therapies to improve KIRC patient outcomes. Our findings highlight BCAT1’s crucial role in KIRC invasion and metastasis.

## Data Availability

Publicly available datasets were analyzed in this study. This data can be found here: GSE159115 https://pubmed.ncbi.nlm.nih.gov/34099557/.

## Supplementary material

Supplementary File 1 Uncropped images of blots of **Figures 4D**, **6A, 6B**, **7A and 7B**.

Supplementary File 2 All 20 pairs of immunohistochemical stained images.
