# Supplementary figures and images for: Identify BCAT1 plays an oncogenic role and promotes EMT in KIRC via single cell RNA-seq and experiment
Source: Front Oncol. 2024 Sep 11;14:1446324. doi: 10.3389/fonc.2024.1446324 (PMC11422235; doi:10.3389/fonc.2024.1446324)

**Additional file 1:Uncropped images of blots of Figure 4D,6A,6B,7A and 7B.**

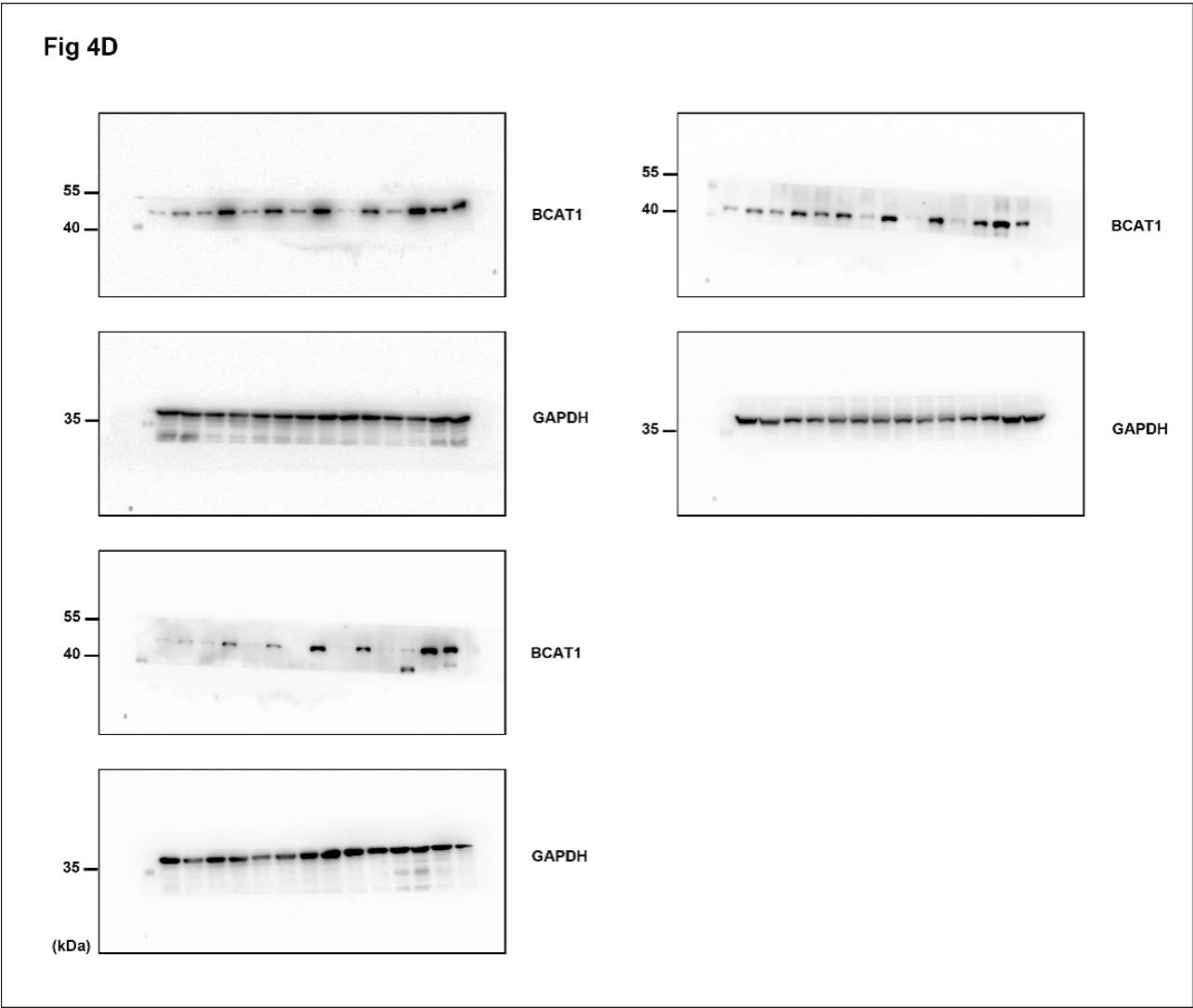

**Fig 6A**

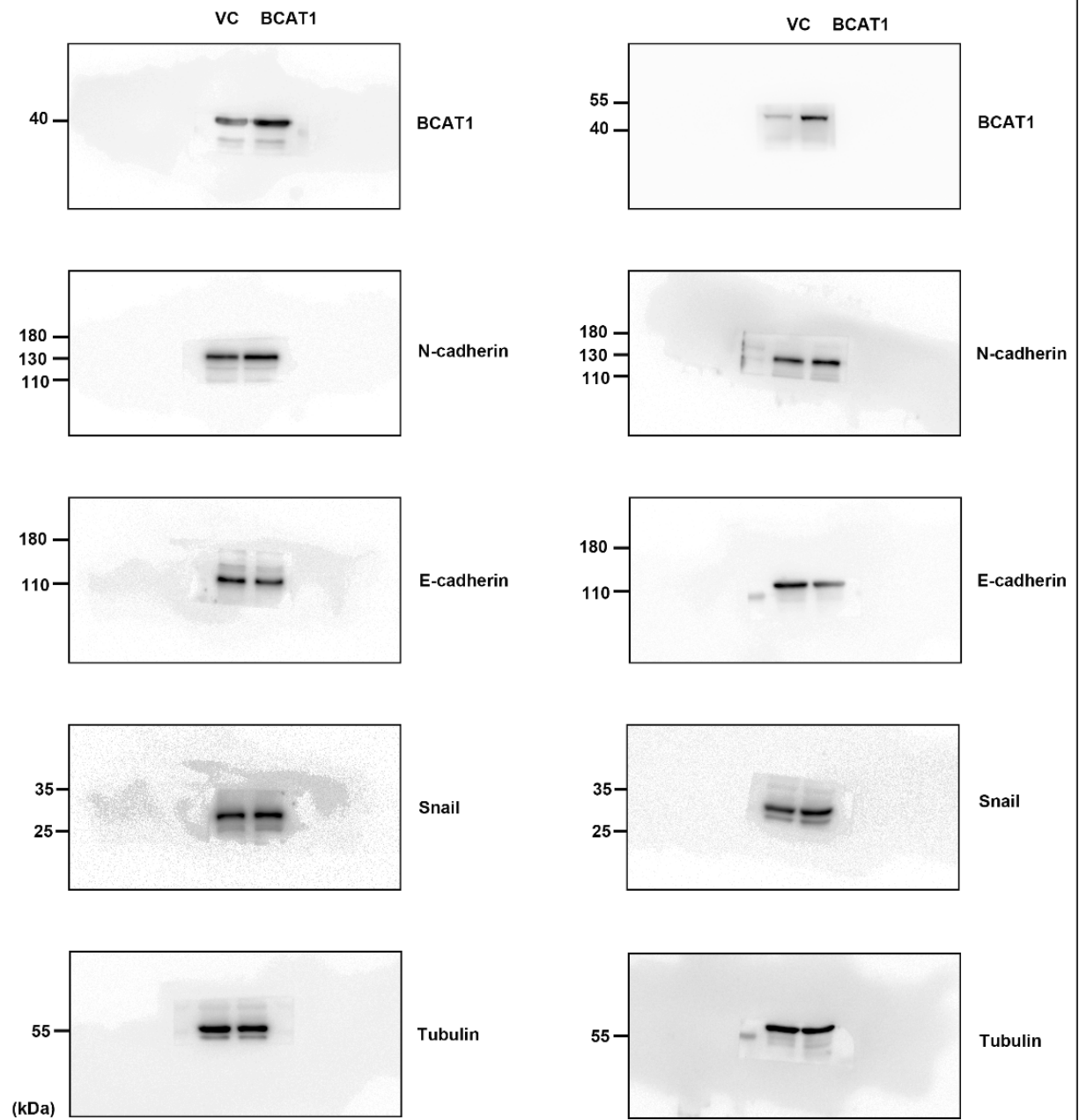

Fig 7A

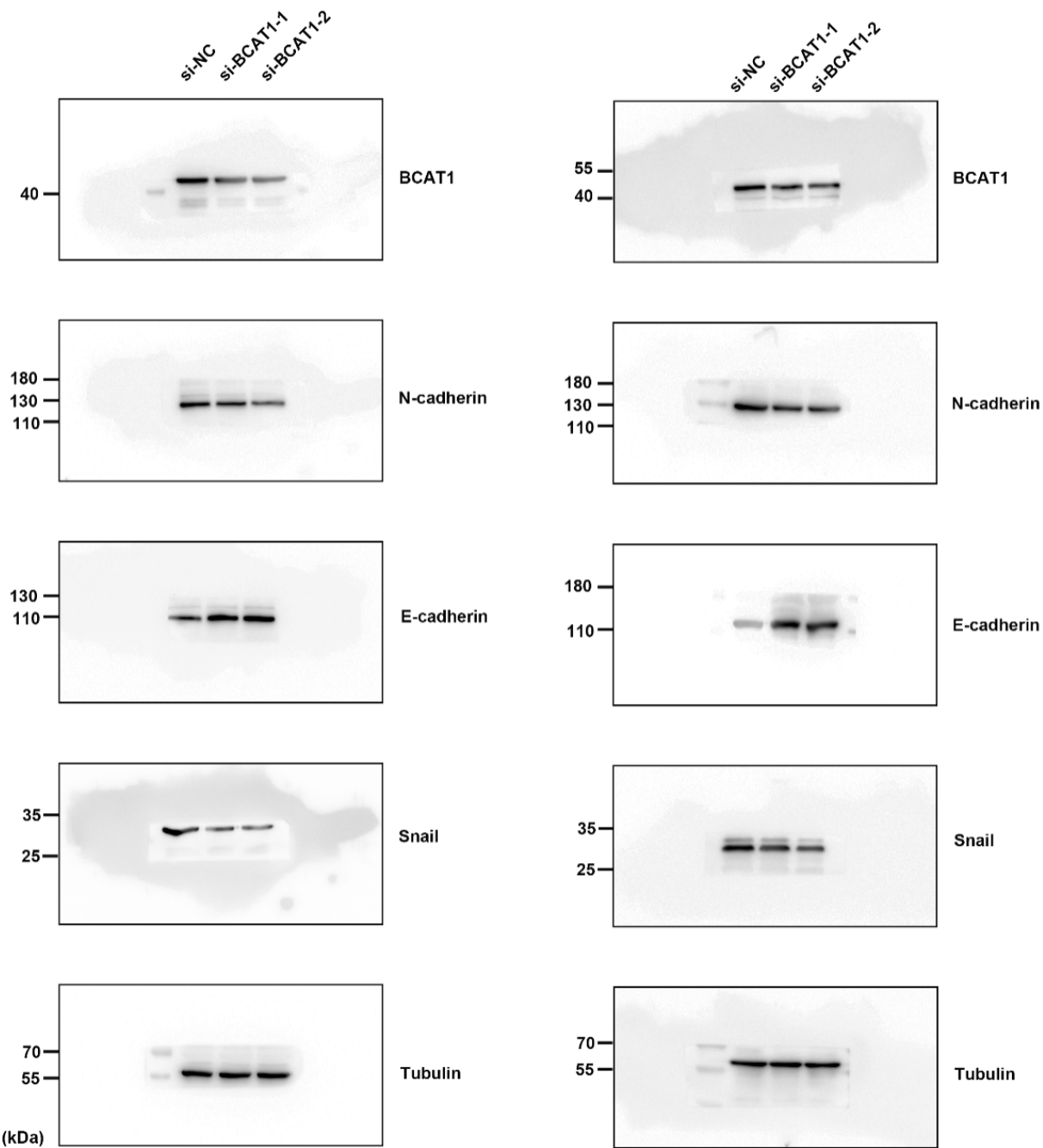

**Fig 6B**

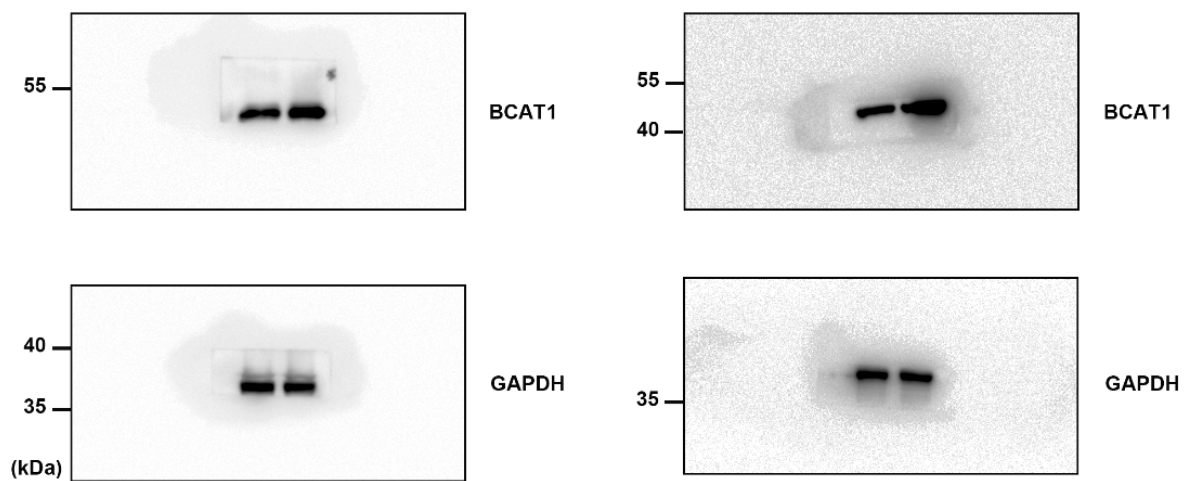

**Fig 7B**

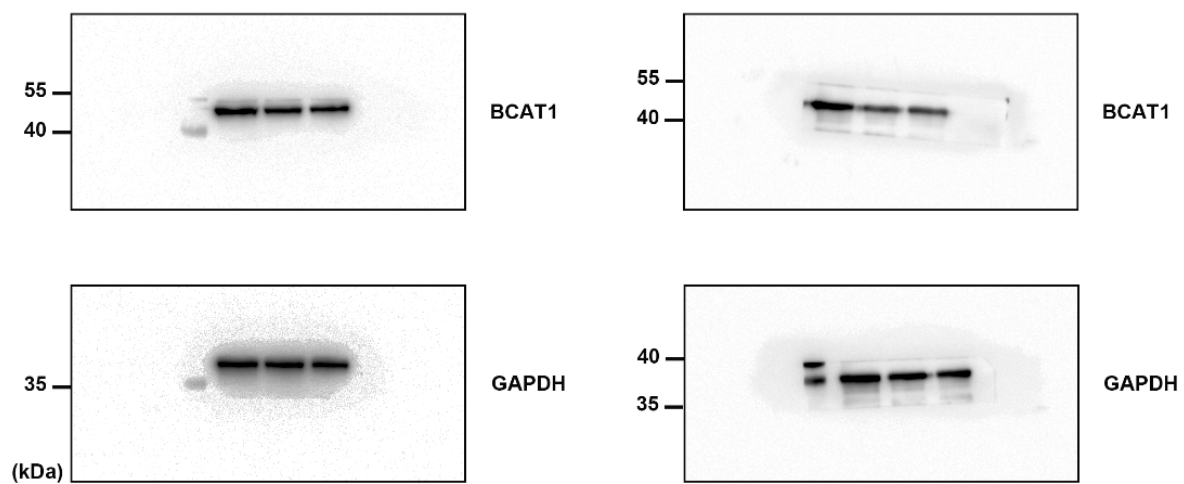

Supplement: Supplementary File 1 — Uncropped images of blots of Figures 4D , 6A, 6B , 7A and 7B . [file DataSheet1.pdf]

Additional file 2: All 20 pairs of immunohistochemical stained images.

N

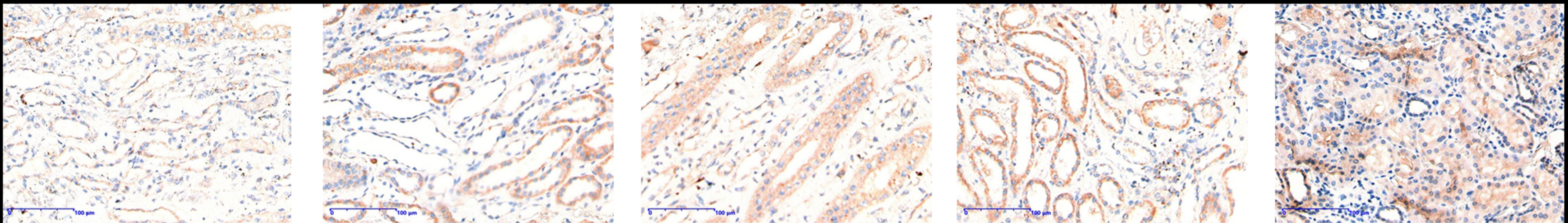

T

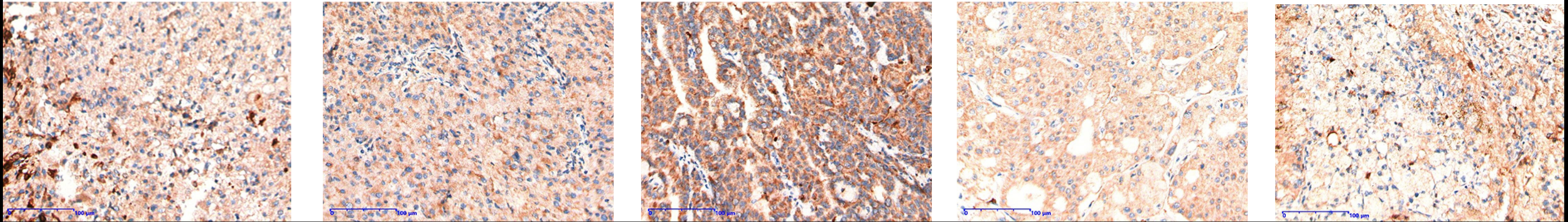

N

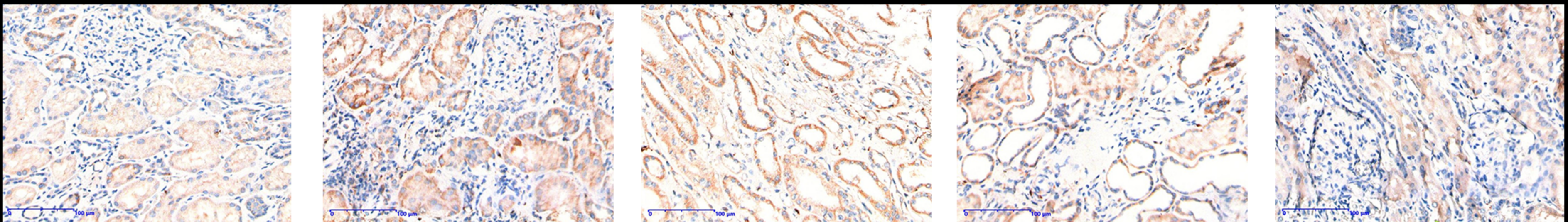

T

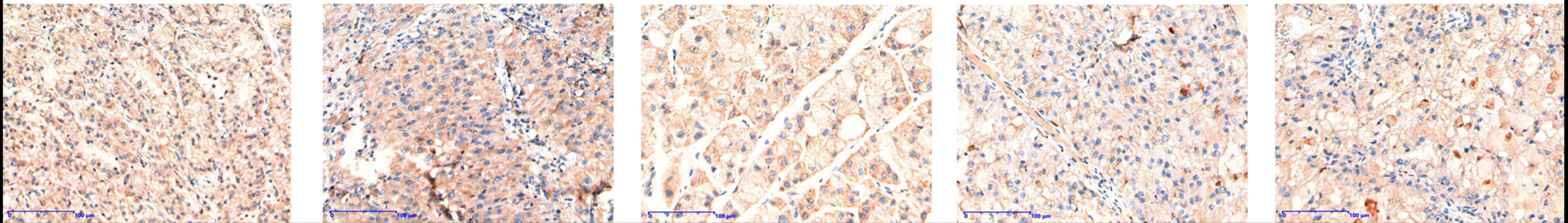

N

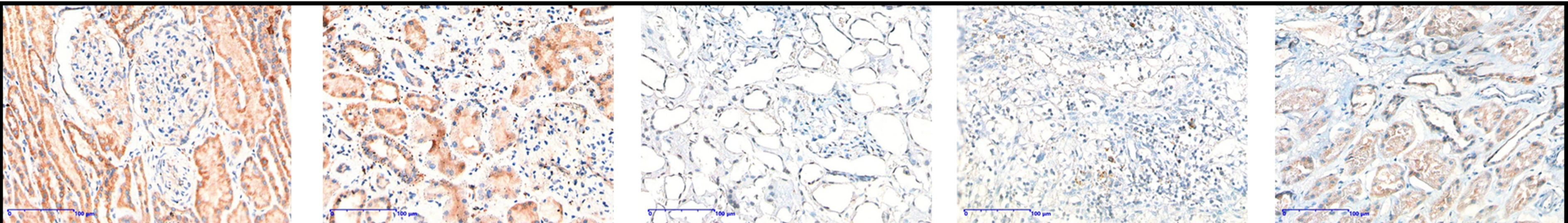

T

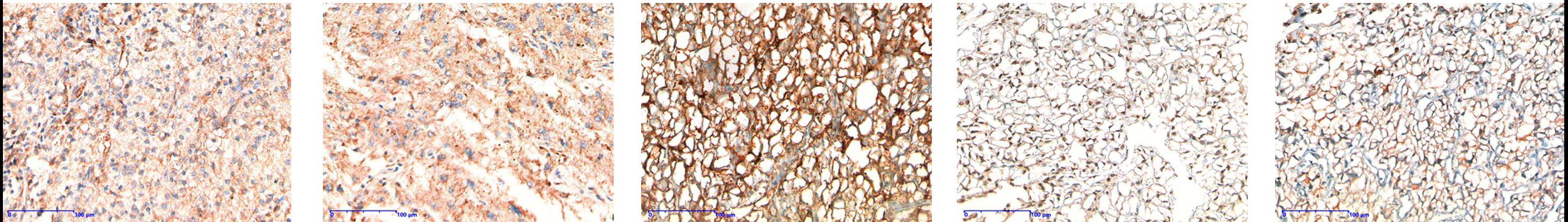

N

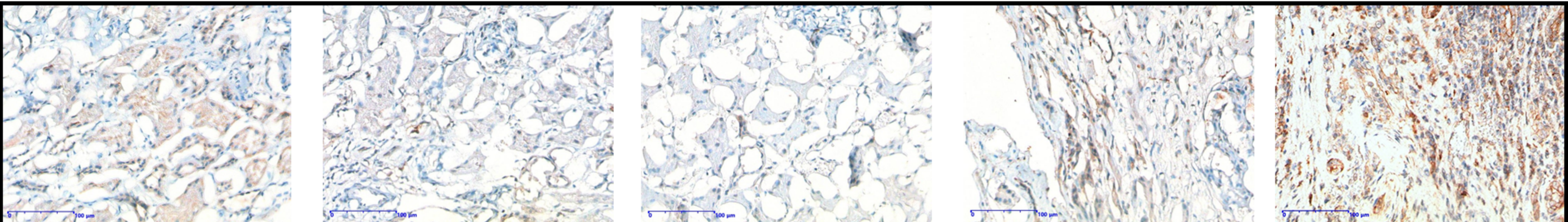

T

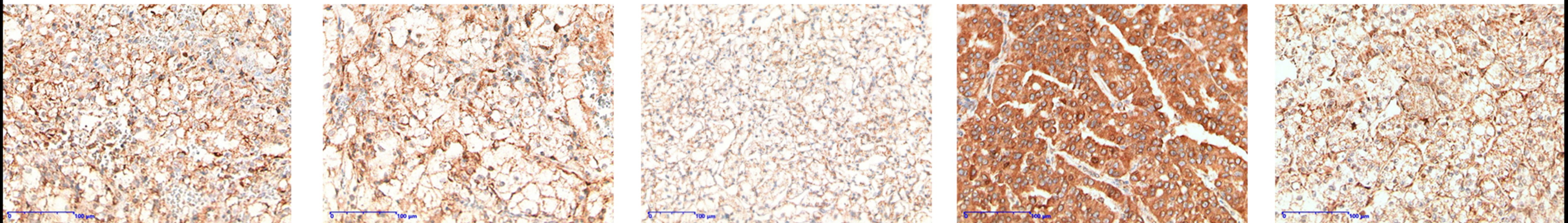

Supplement: Supplementary File 2 — All 20 pairs of immunohistochemical stained images. [file DataSheet2.pdf]
